# Hepatic Sinusoidal Obstruction Syndrome/Veno-Occlusive Disease (SOS/VOD) Primary Prophylaxis in Patients Undergoing Hematopoietic Stem Cell Transplantation: A Network Meta-Analysis of Randomized Controlled Trials

**DOI:** 10.3390/jcm13226917

**Published:** 2024-11-17

**Authors:** Mário Sousa-Pimenta, Ângelo Martins, Letícia M. Estevinho, Carlos Pinho Vaz, Luís Leite, José Mariz

**Affiliations:** 1 Department of Hematology and Bone Marrow Transplantation, Portuguese Oncology Institute of Porto (IPO-Porto), 4200-072 Porto, Portugal; 2 i3S—Instituto de Investigação e Inovação em Saúde da Universidade do Porto, 4200-135 Porto, Portugal; 3 Department of Biomedicine, Unit of Pharmacology and Therapeutics, Faculty of Medicine, University of Porto, 4200-319 Porto, Portugal; 4 Centro de Investigação de Montanha (CIMO), Instituto Politécnico de Bragança, Campus de Santa Apolónia, 5300-252 Bragança, Portugal; 5 Laboratório para a Sustentabilidade e Tecnologia em Regiões de Montanha, Instituto Politécnico de Bragança, Campus de Santa Apolónia, 5300-253 Bragança, Portugal

**Keywords:** hematopoietic stem cell transplantation, sinusoidal obstruction syndrome/veno-occlusive disease (SOS/VOD), prophylaxis, ursodeoxycholic acid, defibrotide, heparin, fresh frozen plasma

## Abstract

**Introduction:** Hepatic sinusoidal obstruction syndrome/veno-occlusive disease (SOS/VOD) is a major complication following hematopoietic stem cell transplantation, resulting from immune and chemical toxicity in the sinusoidal endothelium and hepatocellular damage. In the most severe cases, multiorgan dysfunction occurs, so it is essential to promptly identify patients at greater risk of SOS/VOD and to adopt prophylactic strategies. **Objectives**: This study aims to systematize the impact of different approaches as primary prophylaxes against SOS/VOD in patients undergoing hematopoietic stem cell transplantation (HSCT). **Methods**: A systematic review and meta-analysis of randomized clinical trials evaluating different strategies for primary prophylaxis of SOS/VOD was carried out in pairwise fashion and with a consistent network structure. The odds ratio (OR) and corresponding confidence intervals were calculated using the random-effects model. Heterogeneity was assessed by the I^2^ method and the efficacy of each approach was estimated by SUCRA (surface under the cumulative ranking curve). **Results**: Considering all patients undergoing HSCT, ursodeoxycholic acid (UDCA) [OR = 0.38, 95%CI 0.14–1.06, SUCRA = 0.720] was associated with a lower incidence of VOD while defibrotide reached a modest reduction in its incidence [OR = 0.64, 95%CI 0.23–1.67; SUCRA = 0.486]. Considering the subgroup of patients undergoing hematopoietic progenitors allotransplantation, defibrotide scored higher [OR = 0.51, 95%CI 0.09–2.85, SUCRA = 0.650] by comparison with UDCA [OR = 0.53, 95%CI 0.14–1.96, SUCRA = 0.639]. **Conclusions**: This is the first meta-analysis comparing primary prophylaxes against SOS/VOD. UDCA yielded more promising results when considering all patients undergoing hematopoietic stem cell transplantation, yet, in a subgroup analysis of the ones exposed to allogeneic grafts, it becomes not significantly overrun by defibrotide.

## 1. Introduction

Sinusoidal obstruction/veno-occlusive syndrome (SOS/VOD) associated with hematopoietic stem cell transplantation has an incidence that ranges from 5% [1] to 20%, being tendentiously higher in infants and young adults [2]. Its pathophysiology is representative of the conditioning regimen off-target toxicity, namely endothelial dysfunction, due to the damage inflicted upon endothelial cells and hepatocytes from zone 3 of the hepatic acinus. Briefly, chemotherapeutic agents and radiotherapy induce the activation and damage of endothelial cells, while the fenestration of this barrier promotes the translocation of blood figurate elements and cellular debris towards the space of Disse. These morphophysiological changes and the translocation of microbial-derived products coupled with the immune-reconstitution paradox after hematopoietic stem cell transplantation may further exacerbate sinusoidal and acinar changes [3]. On the other hand, the secretion of cytokines and vasoactive mediators triggers the activation of the coagulation cascade, with the deposition of clotting factors (von Willebrand factor, factor VIII, and fibrin) and vascular tamponade, further obstructing sinusoidal flow [4]. Ultimately, severe SOS/VOD represents a potentially life-threatening condition, evolving from post-sinusoidal portal hypertension and liver dysfunction to multi-organ failure and death [5].

This complication is more likely to occur when certain factors are present during allogeneic hematopoietic stem cell transplantation. These factors include using donors who are not related or have mismatched HLA, using intensive conditioning treatments like busulfan-based regimens or total-body irradiation, and using grafts that have not undergone T-cell depletion. Similarly, the presence of metabolic syndrome, iron overload, pre-existing hepatopathies (cirrhosis, history of viral hepatitis), or hemoglobinopathies (thalassemia) also increases the risk of SOS/VOD [6], as well as previous exposure to therapies with highly hepatotoxic agents, as in the case of gemtuzumab ozogamicin [7] and inotuzumab ozogamicin [8]. Other factors impacting general hemostatic and redox balances also favor SOS/VOD, which itself leads to the consumption of natural circulating anticoagulants such as protein C, S, and antithrombin III [9,10]. Heparanase, an endoglycosidase that cleaves heparan sulfate (HS), also accounts for the burden of disease, with some polymorphisms significantly increasing the risk of SOS/VOD in the first 100 days after allogeneic HSCT [11].

The definition of SOS/VOD has evolved over time, ultimately relying on clinical and analytical parameters. The modified Seattle criteria define SOS/VOD occurring in the first three weeks after HSCT when at least two of the following criteria are present: bilirubin > 2 mg/dL; hepatomegaly and/or right upper abdominal quadrant pain; and weight gain of at least 2% by comparison with baseline [12]. The Baltimore criteria diagnose SOS/VOD when in the first 21 days post-HSCT the serum bilirubin is higher than 2 mg/dL, and at least two other clinical findings occur, namely, hepatomegaly, ascites, or weight gain (over 5% by comparison with pre-HSCT status) in the absence of an alternative medical explanation [13]. However, classical definitions of SOS/VOD do not contemplate the cases diagnosed beyond the first 21 days after HSCT, which, although rarer, are now defined as late-onset SOS/VOD. The European Society for Blood and Marrow Transplantation revised criteria adopted the Baltimore definition for classical SOS/VOD and, in patients beyond 21 days after HSCT, admitted that it can be defined histologically or when there is evidence of reduced or reversed portal flow coupled with at least two additional criteria (bilirubin ≥ 2 mg/dL, painful hepatomegaly, ascites, or weight gain higher than 5% from baseline) [14].

In this review and meta-analysis, we delve into the randomized clinical trials (RCTs) exploring primary prophylactic strategies for SOS/VOD in patients who underwent HSCT. By pooling data retrieved from different RCTs, we ranked the efficacy of different strategies, herein proposing a hierarchization of therapeutic strategies to be adopted according to the patient’s physiological reserve and transplantation protocol.

## 2. Materials and Methods

### 2.1. Literature Search

The research protocol adhered to the Cochrane collaboration guidelines for systematic reviews and PRISMA guidelines [15]. The search was performed in MEDLINE (https://pubmed.ncbi.nlm.nih.gov), SCOPUS (https://www.scopus.com), and Web of Science (https://www.webofknowledge.com). All relevant data were gathered from the inception of the databases up to 16 September 2023, using the following medical subject heading terms: [“Hepatic Veno-Occlusive Disease” or “Sinusoidal obstruction syndrome”] and [“prophylaxis” or “prevention”]. The reference lists of the included studies were analyzed to search for additional studies.

### 2.2. Eligibility Criteria

We aimed to identify all relevant publications focusing on RCTs assessing the efficacy of strategies as primary prophylaxes of SOS/VOD in patients undergoing hematopoietic stem cell transplantation. Only scientific publications that fulfilled the following inclusion criteria were analyzed: (1) RCTs addressing primary prophylaxis regimens directed to SOS/VOD in HSCT recipients; (2) studies including patients who had initiated the therapeutic regimen before or at least concomitantly with the conditioning regimen; and (3) studies reporting the incidence of SOS/VOD.

The exclusion criteria were the following: being a non-randomized clinical trial, post hoc analysis comparing therapeutic interventions started after conditioning initiation, being an observational study, case reports, narrative reviews, experimental studies in basic science/translational domains, guidelines, editorials, correspondences, and consensus/expert statements.

### 2.3. Data Collection

Two authors conducted separate reviews of titles and abstracts from studies identified through electronic searches, excluding those that did not clearly meet the eligibility criteria. The full texts of the remaining articles were then assessed to decide their inclusion or exclusion, with the list of studies selected for inclusion by each author compared and disagreements solved by discussion until consensus.

The following information was abstracted from each study into a data extraction form: type of transplant and conditioning regimen, SOS/VOD defining criteria, and incidence of SOS/VOD in different treatment groups. Differences in data extraction were settled by consensus.

### 2.4. Quality Assessment

Each study’s methodology and reporting quality were assessed independently by authors using the Critical Appraisal Skills Programme (CASP) checklist for RCTs. Furthermore, an analysis of potential bias across the studies was performed by examining funnel plots to identify any signs of asymmetry.

### 2.5. Statistical Analysis

Only RCTs were included in this meta-analysis. Placebo and best supportive care arms were considered as standard care treatments in our analysis, given the absence of any further pharmacological interventions approved for this purpose.

Firstly, a pairwise meta-analysis was conducted comparing the prophylactic interventions following a random-effects model to estimate the pooled OR (odds ratio) and 95% confidence intervals (CI). Statistical heterogeneity was assessed by the I^2^ method. Secondly, a network meta-analysis operated under a consistency model using the Markov chain Monte Carlo (MCMC) method was performed, combining direct and indirect evidence for any given pair of treatments. After model optimization and non-informative prior withdrawal, posterior distributions were obtained using 3.5 × 10^5^ iterations after 10 × 10^4^ burns, with a thinning interval of 5. By comparing the odds of SOS/VOD development under each therapeutic regimen with the standard of care and counting the proportion of iterations in the MCMC method in which each intervention had the lowest OR, we ranked treatments following the SUCRA (surface under the cumulative ranking curve) method. The higher the SUCRA, the higher the efficacy of the intervention [16].

The pooled analysis was performed considering all patients submitted to hematopoietic stem cell transplantation and thereafter was followed by a subgroup analysis considering only the patients submitted to allogeneic hematopoietic stem cell transplantation (either treated with related or unrelated donors’ grafts).

Statistical analyses were performed using the netmeta [17] and GEMTC [18] packages for R, version 3.5.3 (R Foundation for Statistical Computing, Vienna, Austria). A *p*-value less than 0.05 was considered statistically significant and 95% confidence intervals were traced whenever possible to assess significance.

## 3. Results

### 3.1. Selection of Studies and Quality Analysis

**Figure 1:**
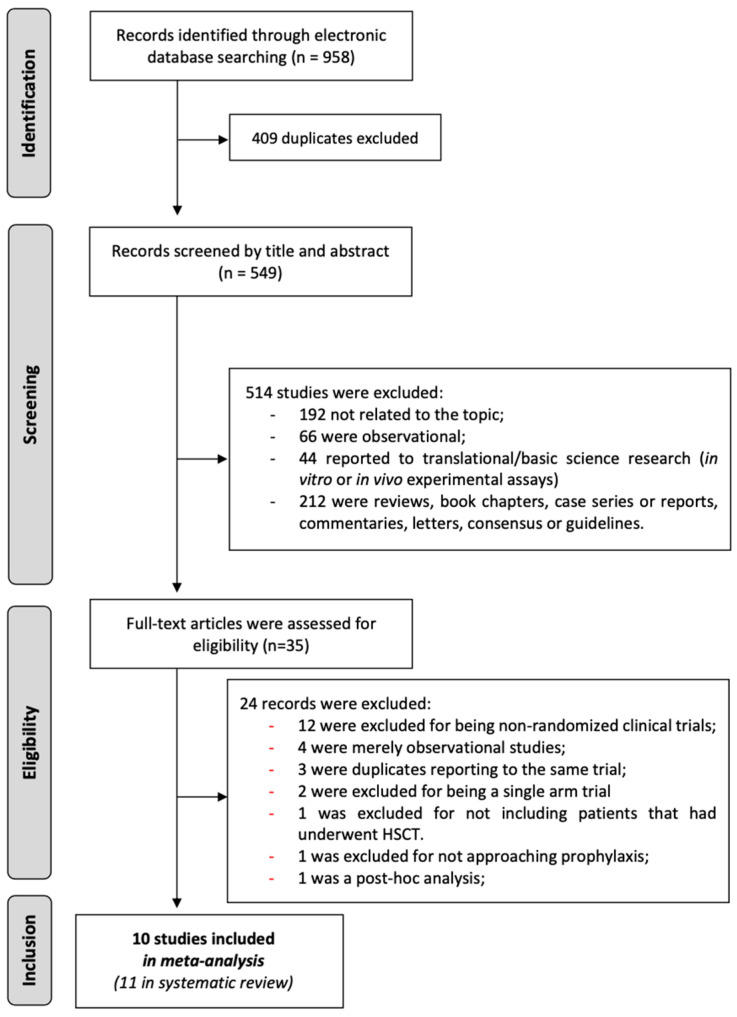
Flowchart of data selection.

After removing duplicates, our search yielded 549 articles. These articles were evaluated based on their titles and abstracts, leading to the exclusion of those that did not meet the inclusion criteria. Subsequently, 35 articles underwent a comprehensive evaluation, resulting in the inclusion of 11 studies in the systematic review and 10 in the meta-analysis. (Figure 1). Among all studies included in the systematic review, ten compared a specific therapeutic intervention with placebo or best supportive care, while one specific trial compared heparin to heparin in association with UDCA.

The overall quality of studies was classified as moderate based on the Comprehensive Assessment of Study Protocols (CASP) for clinical trials amendment (Figure S1, Supplementary Materials). Publication bias was excluded by visual inspection of funnel plots (Figure S2, Supplementary Materials).

### 3.2. Selection of Studies and Quality Analysis

Table 1 summarizes the characteristics of RCTs approaching primary prophylactic regimens for SOS/VOD in the context of hematopoietic stem cell transplantation. Overall, eleven studies were included in the systematic review and ten in the meta-analysis (of which nine were eligible for the consistency model). Concerning the studies included in the meta-analysis protocol, four approached the role of ursodeoxycholic acid [19,20,21,22], two the role of heparin [23,24], one the role of fresh frozen plasma [25], two the role of defibrotide [26,27], and one the role of N-acetyl-L-cysteine [28]. Overall, the studies included in the meta-analysis aggregate the data of 1795 patients and six different therapeutic approaches. In the glycyl-L-glutamine trial [29], there was an absence of events in both treatment and control groups.

The trial addressing the primary prophylaxis with fresh frozen plasma (FFP), while evaluated in the pairwise comparison, was withdrawn from the Bayesian analysis given the absence of events in the experimental arm. The decision was made given that such a finding would lead to an overestimation of FFP efficacy [$\underset{\mathrm{Odds FFP}\to 0}{\lim}\left(\frac{Odds\;prophylactic\;comparator}{Odds\;FFP}\right)=+\text{∞}$], a finding corroborated by the model fit optimization protocol according to the leverage versus square root of residual deviance plot (Figure S3, Supplementary Materials).

Considering all patients that had undergone hematopoietic stem cell transplantation (either allogeneic or autologous), network estimates were pooled considering direct and indirect evidence. Although for five comparisons the pooled estimates are retrieved entirely by direct evidence, ten indirect comparisons are made and entirely based on indirect evidence. The comparator arms UDCA versus UDCA plus heparin, N-acetyl-L-cysteine versus UDCA plus heparin, and defibrotide versus UDCA plus heparin must be interpreted with caution given a mean path length in network estimate higher than two (Figure S4, Supplementary Materials).

A subgroup analysis including only patients with allogeneic transplantation of hematopoietic progenitors pooled the data of 759 patients. The former count excluded patients enrolled in the trial comparing heparin versus UDCA plus heparin, which due to node splitting in our network model induced an impossibility of either directly or indirectly comparing it with other prophylactic approaches; we thus excluded this study from the meta-analysis. Three network estimates were retrieved from direct evidence and the other three from indirect ones. No mean path lengths higher than two were retrieved in network estimates using the random-effects model (Figure S5, Supplementary Materials).

### 3.3. Outcomes of Different Prophylactic Interventions

The pooled data of the odds of SOS/VOD development in patients undergoing HSCT (autologous or allogeneic) were analyzed in pairwise comparison fashion, taking into consideration the direct comparisons and the confidence intervals for the odds traced. Thereafter, direct and indirect comparisons were pooled, and data summarized in a netrank approach taking into consideration the SUCRA indexes evaluated under a consistency model. A subgroup analysis including only patients undergoing allogeneic HSCT was performed.

#### 3.3.1. Patients Undergoing Allogeneic or Autologous Hematopoietic Stem Cell Transplantation

**Figure 2:**
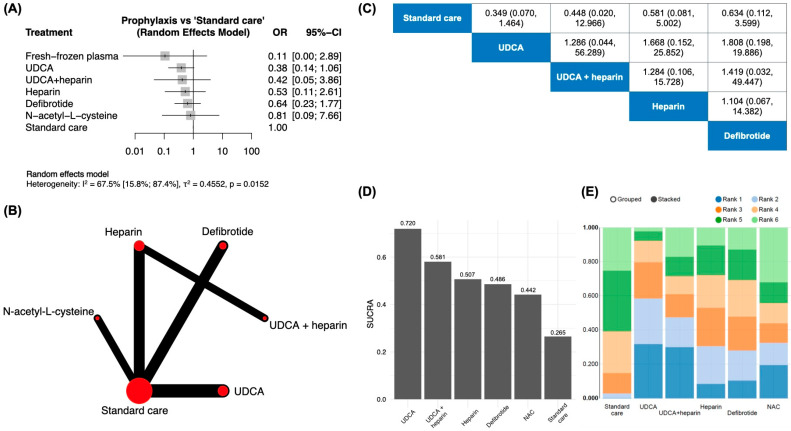
Pooled analysis including all patients undergoing HSCT and enrolled in a clinical trial exploring the efficacy of a prophylactic regimen for SOS/VOD. (**A**)—pairwise meta-analysis addressing the direct comparisons included in the evidence; (**B**)—network analyzed under a consistency model; (**C**)—odds ratio of included interventions and 95% CI (effect of the column-defining intervention relative to the row-defining intervention); (**D**)—SUCRA plots; (**E**)—*rankogram* of prophylactic strategies.

When considering all patients exposed to cell-based therapies (Figure 2, n = 1795), prophylaxis with fresh frozen plasma was associated with the lowest odds of SOS/VOD development in the pairwise comparison [OR of 0.11; 95% CI: 0.00–2.89].

UDCA exhibited an OR of 0.38 [95% CI: 0.14–1.06] and this result was not significantly different from that obtained for the association of UDCA and heparin, which exhibited an OR of 0.42 [95% CI: 0.05–3.86].

A network meta-analysis considering the five direct comparisons and ten indirect comparator arms allowed the estimation of different primary prophylaxis strategies’ efficacy through a cumulative ranking probability index. SUCRA indexes were retrieved and were generally in agreement with the evidence obtained by the pairwise study. UDCA exhibited a SUCRA index of 0.720 (ranking higher in terms of efficacy), while the defibrotide cumulative probability area under the curve was 0.486 (fourth in the ranking). N-acetyl-L-cysteine was associated with the lowest pooled efficacy (SUCRA of 0.442).

#### 3.3.2. Subgroup Analysis of Patients Exposed to Allogeneic Hematopoietic Stem Cell Transplantation

**Figure 3:**
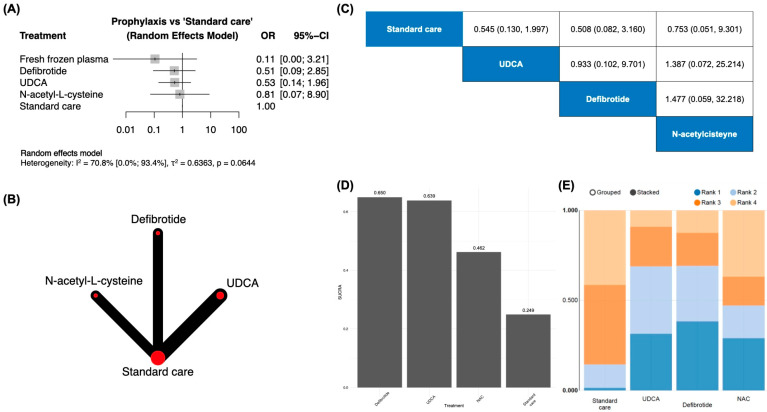
Pooled subgroup analysis including only patients undergoing allogeneic HSCT (both related and unrelated donors included) and enrolled in a randomized trial exploring the efficacy of a prophylactic regimen for SOS/VOD. (**A**)—pairwise meta-analysis addressing the direct comparisons included in the evidence; (**B**)—network analyzed under a consistency model; (**C**)—odds ratio of included interventions and 95% CI (effect of the column-defining intervention relative to the row-defining intervention); (**D**)—SUCRA plots; (**E**)—*rankogram* of prophylactic strategies.

A pairwise analysis in the subgroup of patients treated with allogeneic hematopoietic stem cell transplantation (Figure 3) unveiled the lowest (although not statistically significant) odds of SOS/VOD development in the ones prophylactically treated with fresh frozen plasma, with OR = 0.11 [95% CI: 0.00–3.21].

Although UDCA and defibrotide exhibited similar ORs in the former analysis, a marginally higher SUCRA index was achieved by defibrotide (0.650) in comparison with the UDCA (0.639) when considering the network approach operated under a Bayesian framework. The N-acetyl-L-cysteine prophylaxis ranked once again in the last position (0.462), thus precluding this approach as a potentially effective prophylactic strategy.

## 4. Discussion

As an attempt to examine ways to prevent the development of SOS/VOD, different therapeutic strategies were tested. Ursodeoxycholic acid (UDCA) was shown to enhance biliary secretion of bile acids and bilirubin glucuronides through the post-transcriptional upregulation of export pumps. This agent also exerts anti-apoptotic effects in cholestatic hepatocytes (through a decrease in the mitochondrial release of cytochrome C) and enhances bicarbonate secretion in cholangiocytes (employing an increase in cytosolic calcium that stimulates the chloride/bicarbonate antiporter channel). Interestingly, UDCA displays an immunomodulatory role by decreasing both the expression of MHC (major histocompatibility complex) classes I and II in hepatocytes (the former by a non-selective binding to a glucocorticoid receptor that suppresses the IFN-γ pathway) [30]. Unfractionated heparin, on the other hand, acts through the binding and activation of antithrombin, which ultimately inactivates factor Xa and thrombin, as well as the thrombin-dependent platelet activation and release of factors V and VIII [31]. Concerning the microvascular prothrombotic status and clot formation in hepatic sinusoids and venules [4], heparin may play a role in primary prophylaxis, yet at the expense of an increased bleeding risk. Defibrotide, in another spectrum, is an orphan-drug known for its profibrinolytic activities on the dependence of the increased expression and activity of tissue plasminogen activators, tissue factor pathway inhibitors, and thrombomodulin—coupled with its antithrombotic properties on the dependence of the reduced expression of plasminogen activator inhibitors, platelet-activating factor, and thrombin—ultimately decreasing platelet activation and secondary hemostasis. Defibrotide was also shown to downregulate MHC-I and II expression (the former induced by prostaglandins E2 and I2 increased expression) [32]. Given its master player role in hemostasis, vascular biology, and immune-modulatory properties, defibrotide has been explored not only in the treatment but also as a prophylactic strategy in patients with a higher risk of developing SOS/VOD. Lastly, and considering the tested strategies in former trials, N-acetyl-L-cysteine was explored given its role as a scavenger of reactive oxygen species and glutathione precursor [33]. To date, there is still no consensus on the demand and efficacy of primary prophylactic strategies in SOS/VOD, as well as the target population that could benefit most from this strategy.

In this analysis, we report an aggregated overview of the role of primary prophylaxis of SOS/VOD in patients undergoing hematopoietic stem cell transplantation. Considering all patients undergoing cell-based therapies, UDCA scored higher. However, when only allotransplanted patients were analyzed, defibrotide marginally outperformed UDCA in this subset of patients. This observation, beyond being related to the nature of the procedure (autologous or allogeneic), potentially reflects patient and disease intrinsic characteristics that vary across these two therapeutic procedures. Allotransplanted patients are typically diagnosed with more aggressive neoplasms (acute leukemias, for example) [34]; during induction and consolidation therapies, they are exposed to significant cumulative doses of cytotoxic agents, exhibiting a higher need for blood transfusions that otherwise induce hemosiderosis and increase iron deposits in liver [35]. The conditioning regimens vary widely across studies (and even over time); it is well known that, for example, liver irradiation may potentiate SOS/VOD incidence, as shown in a previous analysis where the incidence of this complication was increased in patients exposed to single-dose versus hyperfractioned regimens [36]. In our analysis, the distribution of allotransplanted patients whose conditioning regimens were dependent upon total body radiation was not uniformly distributed across different trials (which was also the case regarding pre-transplantation needs for irradiation of the abdominal compartment), and this circumstance may impose milder heterogeneity in the findings. Immune reconstitution following allogeneic transplantation of hematopoietic progenitors will also induce cellular damage, depending on the degree of HLA (human leukocyte antigen) matching [37], another observation that may impose undetermined heterogeneity on the pooled population. Although a multivariate analysis to assess the role of conditioning regimen intensity, exposure to total body irradiation, protocols employed for GVHD prophylaxis, or certain immunochemotherapeutic strategies would be highly desirable, the lack of available data made such an analysis unfeasible. The same issue arises with subgroup analysis regarding the incidence of SOS/VOD in pediatric patients compared to older ones: there are fewer trials focused solely on pediatric patients, and a lack of subgroup outcomes information in those that enroll patients regardless of age.

In light of current knowledge, it is fair to hypothesize that the minimal advantage of primary prophylaxes with defibrotide in allotransplanted patients may arise from its immunomodulatory and iron chelation properties [32], which secondarily reduce oxidative stress and inflammation associated with iron-induced liver disease. In fact, elevated serum ferritin levels before hematopoietic stem cell transplantation are regarded as an important risk factor for SOS/VOD development [38]. Pre-transplantation transfusion dependency (associated with hemosiderosis) was associated with lower overall survival, a higher burden of graft-versus-host disease, and an increased non-relapse mortality in patients undergoing myeloablative conditioning regimens [39], with therapeutic strategies to deplete iron stores in historical cohorts even showing an increase in liver function tests of patients after bone marrow transplantation [40]. Notwithstanding this potential explanation, it is important to take into consideration that UDCA and defibrotide are the main strategies that have been tested as primary prophylactic regimens. Beyond their over-representation, in a defibrotide trial, UDCA was even allowed in both arms [26].

Beyond the patient, disease, and therapeutic measures’ intrinsic properties that may influence the hazard of SOS/VOD development, observational bias in clinical trials may also limit the extrapolation of general conclusions drawn from them. For instance, recent trials were pivotal by showing that there was a disagreement in SOS/VOD diagnosis between local physicians (thoroughly accompanying the patient journey) and the ones responsible for centrally and blindly revising clinical and analytical data [27]. The dependence of the diagnosis of this entity on clinical and analytical criteria (both dynamic) may undoubtedly influence its diagnosis and reported incidence.

Overall, when considering the pharmacological agents assessed in our analysis, ursodeoxycholic acid, beyond being highly affordable, displays a pleiotropic pharmacological action, modulating both liver bile acids secretion, hepatocyte resistance to apoptosis and decreasing the potential for immune-mediated injury by downregulating MHC expression on the surface of liver cells [30]. For these reasons, evidence supports its use as a beneficial prophylactic strategy in patients undergoing hematopoietic stem cell transplantation. Although it was initiated before or at least concomitantly with the conditioning regimens in the analyzed trials, it is reasonable to hypothesize that starting even earlier (particularly in patients exposed to immunochemotherapeutic strategies, such as antibody–drug conjugates) could yield even better results due to its multifactorial pharmacological properties.

When considering only patients undergoing allogeneic bone marrow transplantation, we systematize and report, for the first time, an aggregated overview of the primary prophylactic strategies for SOS/VOD. Despite the population heterogeneity, which could not have been avoided in the pooled analysis, taking into consideration our findings, the pharmacological principles of the different therapeutic strategies, and the immune (dis)regulation associated with each particular transplant, we have opened doors to improved critical thinking and decision making in clinical practice by proposing a hierarchization of therapeutic strategies.

## 5. Conclusions

The adoption of primary prophylactic regimens for SOS/VOD in patients undergoing hematopoietic stem cell transplantation remains a controversial issue. The diversity of pathologies imposing the need for cell-based therapies, together with the heterogeneity of conditioning regimens and patients’ basal immunological fitness and immune reconstitution after stem cell engraftment, impose some limitations when it comes to generalizing the observations coming from clinical trials.

Our network meta-analysis revealed that UDCA is an overall promising strategy, considering both patients undergoing autologous or allogeneic hematopoietic stem cell transplantation. A subgroup analysis in allotransplanted patients (made whenever specific data about this group were available and retrievable from clinical trials) also supported defibrotide as a promising strategy in this context. It is expected that some patients (we hypothesize the ones with liver iron overload, for example) may benefit from defibrotide instead of UDCA. Notwithstanding this, due to its accessibility and affordability, UDCA seems to be a promising baseline strategy, particularly in lower- to middle-income countries. Further studies are warranted to identify the patients who will benefit more from primary prophylaxes, as well as to systematize the clinical and biological markers that indicate the need for a specific therapeutic approach.

## Figures and Tables

**Table 1:** Summary of randomized clinical trials assessing the outcome of prophylactic regimens in SOS/VOD.

Intervention	Cell Therapy	Population	Intervention	Control	SOS/VOD Criteria	Ref.
**UDCA**	AlloHSCT (related donor)	Total of 67 patients undergoing allogeneic (related donor) HSCT, conditioned with busulfan plus cyclophosphamide; exposed to GVHD prophylaxis with cyclosporine plus methotrexate.	UDCA 300 mg twice daily (or 300 mg + 600 mg if body weight was >90 kg) compared with placebo.	Placebo	Seattle	[19]
	AlloHSCT AutoHSCT	Total of 56 patients submitted to AlloSCT (related or unrelated/mismatched donor) and 15 to autologous hematopoietic stem cell transplantation were randomized to receive primary prophylaxis with UDCA.	UDCA 600 mg daily, from day −21 up to day +80.	No specific treatment	Seattle	[20]
	AlloHSCT AutoHSCT	Total of 81 patients underwent allogeneic HSCT and 88 autologous HSCT.	UDCA 300 mg twice daily + heparin 5 U/Kg (starting up to 24 h before conditioning) up to day +30.	Heparin	Modified Seattle	[21]
	AlloHSCT	Total of 132 patients receiving allogeneic HSCT from related donors and 110 from matched unrelated donors were randomized to receive primary prophylaxis with UDCA.	UDCA 12 mg/Kg/day from 24 h before starting conditioning regimen up to D + 90 after transplantation.	No specific treatment	Seattle/Baltimore	[22]
**Heparin**	AlloHSCT AutoHSCT	Total of 79 patients undergoing allogeneic HSCT (non-T cell depleted) and 81 undergoing autologous HSCT were randomized to receive heparin.	Heparin (100 U/kg/day) in continuous infusion from day −8 up to day +30 after HSCT.	No specific treatment	Seattle	[23]
	AutoHSCT	Total of 92 patients undergoing autoHSCT (without criteria for high risk of VOD) randomized to receive heparin.	Heparin at 1 mg/Kg in continuous infusion by day 0 until recovery or discharge.	No specific treatment	Seattle	[24]
**Fresh frozen plasma**	AlloHSCT	Total of 43 patients (15 children) undergoing alloHSCT with high risk of developing VOD (exposed to intensified conditioning regimens, undergoing second SCT, or with prior liver dysfunction) were randomized.	FFP twice weekly and up to day + 28 of HSCT according to body weight: 1 U (80 mL) if <10 kg, 2 U if 10–20 kg, 3 U if 20–30 Kg, 4 U if 30–40 kg or 5 U if >40 Kg.	No specific treatment	Seattle	[25]
**Defibrotide**	AlloHSCT AutoHSCT	Patients < 18 years with risk factors for VOD, undergoing allogeneic or autologous HSCT; UDCA allowed in both arms.	Defibrotide 25 mg/kg/day starting with conditioning regimen up to day + 30 after HSCT.	No specific treatment	Modified Seattle	[26]
	AlloHSCT AutoHSCT	Adult (>16 years) and pediatric patients (<16 years) receiving alloHSCT or autologous HSCT (the former only in pediatric) with high risk of developing SOS/VOD were randomized to receive defibrotide.	Defibrotide 25 mg/kg/day starting with the conditioning regimen up to day + 21 at least and no more than day + 30 after HSCT.	No specific treatment	Modified Seattle	[27]
**N-acetyl-L-cysteine**	AlloHSCT	Patients undergoing allogeneic HSCT were randomized to receive or not NAC if risk factors were present (elevated bilirubin, ALT, or AST). Total of 28 patients were exposed to FTBI (9 with NAC prophylaxis) and 48 to BuCy conditioning (20 exposed to NAC).	NAC infusion of 6 h at dose of 100 mg/Kg/day until normalization of bilirubin, ALT, and AST values.	No specific treatment	Baltimore	[28]
**Glycyl-L-glutamine**	AlloHSCT AutoHSCT	Patients undergoing allogeneic HSCT (n = 7) or autologous HSCT (n = 27). Some patients received warfarin until platelet counts dropped below 50 000/uL (nine in experimental arm and two in control).	Daily infusion of 50 g glycyl-L-glutamine.	Isonitrogenous mixture of non-essential amino acids (50 g/day)	Not specified (incidence of VOD not displayed)	[29]

Legend: BuCy—busulfan plus cyclophosphamide; FTBI—fractionated total body irradiation; GVHD—graft versus host disease; HSCT—hematopoietic stem cell transplantation; NAC—N-acetyl-L-cysteine; SCT—stem cell transplant. Ref—reference.

## Data Availability

All curated data are presented in the main text and Supplementary Files, with sources cited within this review. Any further information will be provided by the authors upon request.

## Supplementary Materials

The following supporting information can be downloaded at: https://www.mdpi.com/article/10.3390/jcm13226917/s1. Figure S1: Critical Appraisal Skills Programme (CASP) checklist for clinical trials; Figure S2: Funnel plots illustrating the meta-analysis results for the included studies in the pooled analysis for all (autologous and allogeneic) patients treated with HSCT and the subgroup analysis with ones treated with allogeneic HSCT; Figure S3: Leverage versus square root of residual deviance plot of the studies included in the network meta-analysis. The outlier pattern of FFP (fresh frozen plasma) on the graph on the left (red circle), further prompted its exclusion from the pooled analysis; Figure S4: Direct and indirect evidence proportions of network estimates according to a random effects model in all patients submitted to hematopoietic stem cells transplantation; Figure S5: Direct and indirect evidence proportions of network estimates according to a random effects model in patients who underwent allogeneic hematopoietic stem cells transplantation (subgroup analysis).
