# Supplementary material for: Hepatic Sinusoidal Obstruction Syndrome/Veno-Occlusive Disease (SOS/VOD) Primary Prophylaxis in Patients Undergoing Hematopoietic Stem Cell Transplantation: A Network Meta-Analysis of Randomized Controlled Trials
Source: J Clin Med. 2024 Nov 17;13(22):6917. doi: 10.3390/jcm13226917 (PMC11594704; doi:10.3390/jcm13226917)
Supplement: Supplementary file 1 [file jcm-13-06917-s001.zip › jcm-3205837-supplementary.pdf]

Supplementary Data:

CASP Checklist - Cohort Study	1	2	3	4	5	6	7	8	9	10	11
Essell et al., 1998	Yes	Yes	Yes	Yes	Yes	Yes	Yes	Yes	Yes	Can't tell	Yes
Ohashi et al., 2000	Yes	Yes	Yes	No	Yes	Yes	Yes	Yes	Yes	Can't tell	Yes
Park et al., 2002	Yes	Yes	Yes	Can't tell	Can't tell	Yes	Yes	Yes	Yes	Can't tell	Yes
Ruutu et al., 2002	Yes	Yes	Yes	Can't tell	Yes	Yes	Yes	Yes	Yes	Can't tell	Yes
Attal et al., 1992	Yes	Yes	Yes	Can't tell	Yes	Yes	Yes	Yes	Yes	Can't tell	Can't tell
Marsa-Vila et al., 1991	Yes	Yes	Yes	No	Yes	Yes	Yes	Yes	Yes	Can't tell	Can't tell
Matsumoto et al., 2007	Yes	Yes	Yes	No	No	Yes	Yes	Yes	Yes	Can't tell	Can't tell
Corbacioglu et al., 2012	Yes	Yes	Yes	No	Yes	No	Yes	Yes	Yes	Can't tell	Can't tell
Grupp et al., 2023	Yes	Yes	Yes	Can't tell	Yes	Yes	Yes	Yes	Yes	Can't tell	Can't tell
Barkholt et al., 2008	Yes	Yes	Yes	No	Can't tell	Yes	Yes	Yes	Can't tell	Can't tell	No
SA et al., 1998	Yes	Yes	Yes	Yes	No	No	Yes	Yes	Can't tell	Can't tell	No

Figure S1: Critical Appraisal Skills Programme (CASP) checklist for clinical trials.

Questions correspondence as follows:

1. Did the study address a clearly focused research question?
2. Was the assignment of participants to interventions randomized?
3. Were all participants who entered the study accounted for at its conclusion?
4. Were the participants 'blind' to intervention they were given? Were the investigators 'blind' to the intervention they were giving to participants? Were the people assessing/analysing outcome/s 'blinded'?
5. Were the study groups similar at the start of the randomized controlled trial?
6. Apart from the experimental intervention, did each study group receive the same level of care (that is, were they treated equally)?
7. Were the effects of intervention reported comprehensively?
8. Was the precision of the estimate of the intervention or treatment effect reported?
9. Do the benefits of the experimental intervention outweigh the harms and costs?
10. Can the results be applied to your local population/in your context?
11. Would the experimental intervention provide greater value to the people in your care than any of the existing interventions?

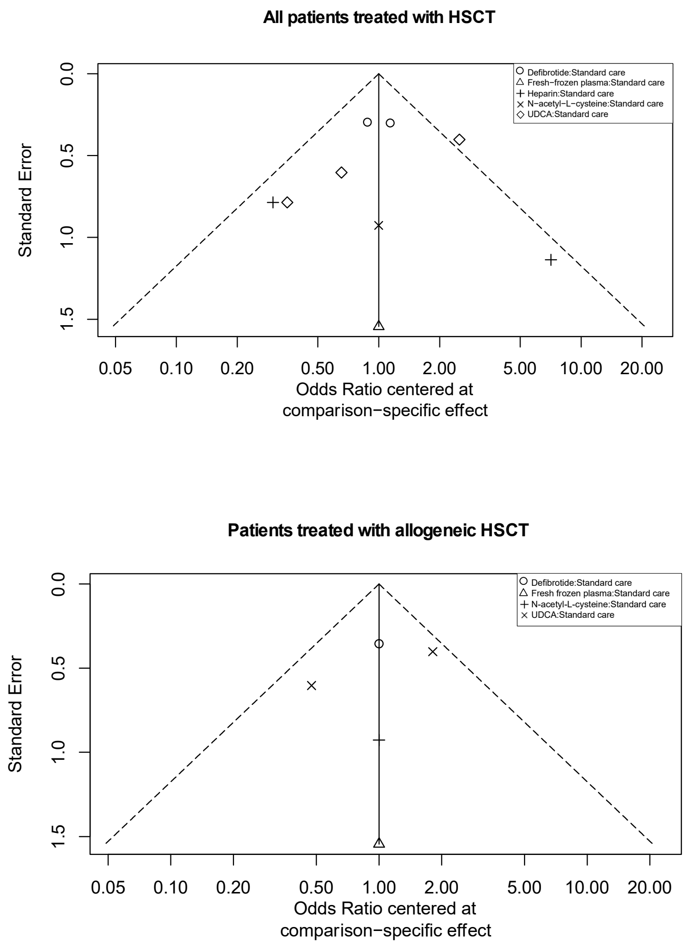

Figure S2: Funnel plots illustrating the meta-analysis results for the included studies in the pooled analysis for all (autologous and allogeneic) patients treated with HSCT and the subgroup analysis with ones treated with allogeneic HSCT.

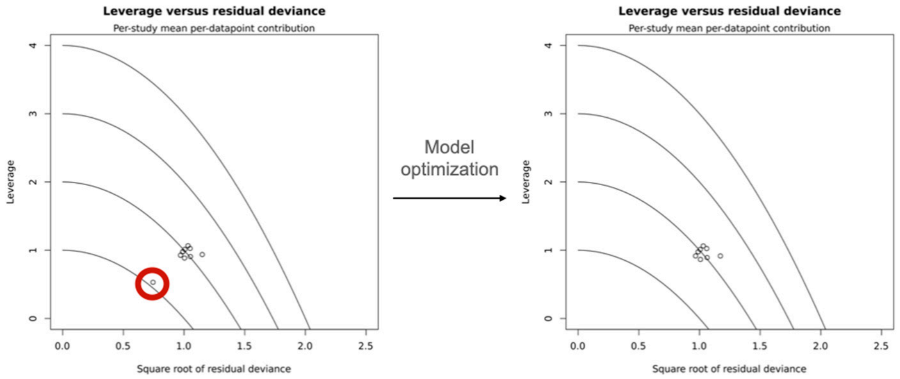

Figure S3: Leverage *versus* square root of residual deviance plot of the studies included in the network meta-analysis. The outlier pattern of FFP (fresh frozen plasma) on the graph on the left (red circle), further prompted its exclusion from the pooled analysis.

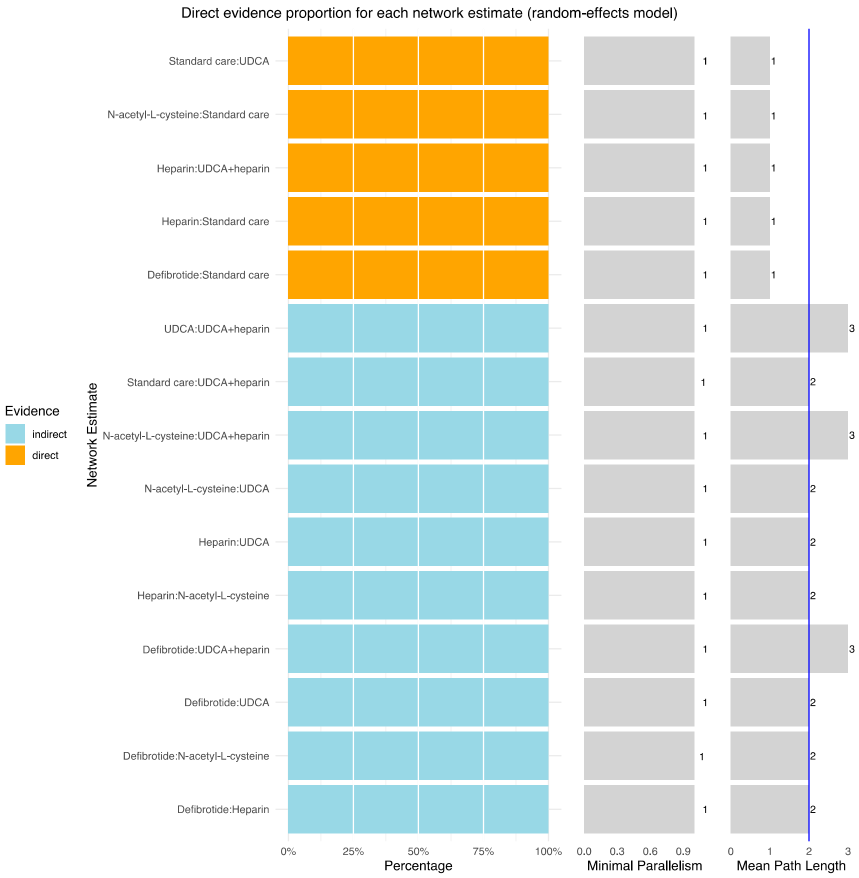

Figure S4: Direct and indirect evidence proportions of network estimates according to a random effects model in all patients submitted to hematopoietic stem cells transplantation.

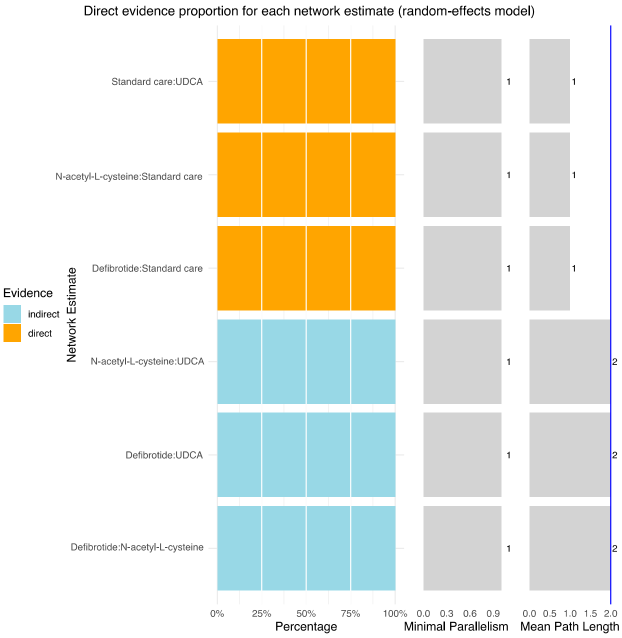

Figure S5: Direct and indirect evidence proportions of network estimates according to a random effects model in patients who underwent allogeneic hematopoietic stem cells transplantation (subgroup analysis).
